# Immune Response in Calves Vaccinated with Type Three Secretion System Antigens and Shiga Toxin 2B Subunit of *Escherichia coli* O157:H7

**DOI:** 10.1371/journal.pone.0169422

**Published:** 2017-01-03

**Authors:** Luisina Martorelli, Sergio Garbaccio, Daniel A. Vilte, Adriana A. Albanese, María P. Mejías, Marina S. Palermo, Elsa C. Mercado, Cristina E. Ibarra, Angel A. Cataldi

**Affiliations:** 1 Instituto de Patobiología, Centro de Investigación en Ciencias Veterinarias y Agronómicas, Instituto Nacional de Tecnología Agropecuaria, Hurlingham, Argentina; 2 Laboratorio de Fisiopatogenia, Departamento de Fisiología, Facultad de Medicina, Universidad de Buenos Aires, Buenos Aires, Argentina; 3 Laboratorio de Patogénesis e Inmunología de Procesos Infecciosos, Instituto de Medicina Experimental, (IMEX), Consejo Nacional de Investigaciones Científicas y Técnicas (CONICET)- Academia Nacional de Medicina, Buenos Aires, Argentina; 4 Instituto de Biotecnología, Centro de Investigación en Ciencias Veterinarias y Agronómicas, Instituto Nacional de Tecnología Agropecuaria, Hurlingham, Argentina; Cornell University, UNITED STATES

## Abstract

Ruminants are the primary reservoir of Shiga-toxin producing *Escherichia coli* (STEC) O157:H7 and the main source of infection for humans. The aim of this study was to assess the immunogenic properties of a candidate vaccine consisting on the recombinant proteins of *E*. *coli* O157:H7 IntiminC_280_, the carboxy-terminal fraction of Intimin γ, EspB and the fusion protein between the B subunit of Stx2 and Brucella Lumazine Synthase (BLS)(BLS-Stx2B), in Holstein Fresian calves.To accomplish this goal we vaccinated calves with two doses of different vaccine formulations: 2 antigens (IntiminC_280_, EspB), 3 antigens (IntiminC_280_, EspB, BLS-Stx2B), BLS-Stx2B alone and a control non-vaccinated group. All antigens were expressed as recombinant proteins in *E*. *coli*. Specific IgG titres increased in vaccinated calves and the inclusion of BLS-Stx2B in the formulation seems to have a stimulatory effect on the humoral response to IntiminC_280_ and EspB after the booster. The neutralizing activity of antibodies against these two antigens was assessed in Red Blood Cell lysis assays and adherence to Hep-2 cells as a correlate of T3SS activity. Both sera from animals vaccinated with 2 or 3 antigens inhibited both virulence properties. Serological response to Stx2 was observed in animals vaccinated only with BLS-Stx2B and with 3 antigens and neutralization of Stx2 cytotoxicity was also observed in both groups. In conclusion, immunization of calves with BLS-Stx2B, IntiminC_280_ and EspB elicited a potent humoral response able to neutralize Shiga toxin 2 cytotoxity and the T3SS virulence properties *in vitro*. These results suggest that this formulation is a good candidate vaccine to reduce STEC shedding in cattle and needs to be further assessed *in vivo*.

## Introduction

Enterohemorragic *Escherichia coli* (EHEC) O157:H7 is a major etiologic agent of diseases in humans, whose clinical spectrum includes diarrhoea, haemorrhagic colitis and haemolytic uremic syndrome (HUS), the leading cause of chronic renal failure in children in Argentina and several other countries[1, 2].

Cattle are the main reservoir of EHEC O157:H7, which predominately colonizes the lymphoid follicle-dense mucosa at the terminal rectum and the rectoanal junction (RAJ)[3].

*E*. *coli* O157:H7 is characterized by several virulence-associated traits which enables it to colonize the intestinal mucosa of humans and animals with a characteristic histopathological lesion known as “attaching and effacing” (A/E). A large chromosomal pathogenicity island called Locus of Enterocyte Effacement (LEE) is associated with A/E activity [4–6]. The LEE encodes a type three secretion system (TTSS) that translocates effector proteins responsible for the A/E lesion into the host cell. Tir, EspB and other LEE-encoded and non-LEE encoded effectors are translocated into the host cell through a transiently produced filamentous structure [7], which consists of an assembly of EspA subunits [8] and contributes, in turn, to the creation of a pore in the eukaryotic cell membrane. Intimin, a bacterial outer membrane protein, binds to Tir, the translocated Intimin receptor in the host cell membrane, and this binding leads to the formation of the A/E lesion.

This bacterium also produces Shiga toxins types 1 and/or 2 [9–11], which are responsible for systemic damage in humans. In cattle, a partial suppression of the mucosal immune response by Shiga toxin has been observed, apparently favouring the intestinal colonization by *E*. *coli* O157:H7 [12–18].

Many virulence factors of *E*. *coli* O157: H7 induce an immune response during the course of natural or experimental infections in animals and in patients with HUS. Oral inoculation of calves and steers with *E*. *coli* O157: H7 promotes an increase in serum antibody titres against O157 lipopolysaccharide and neutralizing antibodies to Shiga toxins [19]. Furthermore, Bretschneider et al [20] demonstrated that cattle respond serologically to Intimin and EspB of *E*. *coli* O157:H7 during the course of experimental infection. Antibodies against these proteins have also been detected in colostra and milk from cows [21–23]

Several authors have reported that calves and adult cattle shed fewer bacteria after several experimental inoculations, which could be related to a partially protective immune response elicited by previous infection [24–27]. Our group has demonstrated that naturally acquired antibodies against IntiminC_280_ can reduce shedding in experimentally challenged calves, suggesting a protective role for antibodies [27, 28].Vaccination of cattle with bacterial colonization factors has been suggested as a strategy to prevent *E*. *coli* O157:H7 infection. Various vaccine formulations have been assayed with variable results [29–34]. We, along with other groups, have demonstrated that vaccination of calves with type three secretion injection apparatus proteins results in reduced excretion of EHEC O157:H7 after experimental infection with an oral challenge dose of 10^10^ CFU [29, 32–35]. Despite the reduced shedding observed, protection was not complete and thus, the current vaccination strategy is ought to be optimized.

As mentioned above, Stx might act as an immunomodulating agent during STEC infections in cattle and is a virulence factor harboured by all STEC strains, which makes them interesting vaccine candidates [36]. Considering that Stx2 is the most pathogenic Stx toxin[37], we chose a Stx2B-based immunogen to raise antibodies against Stx2. Taking into account that its B subunit is a very poor immunogen[38], a novel antigen which comprises the B subunit of Stx2 fused to the N-terminus of Brucella Lumazine Synthase (BLS) was used [39]. This highly stable BLS-Stx2B fusion protein was able to induce a significant response in mice [40] and therefore we tested this immunogen in cattle.

In consequence, the aim of this study was to assess the immunogenic properties of BLS-Stx2B, and the effect of the inclusion of this antigen on the response to IntiminC_280_ and EspB, as well as to evaluate the ability of the antibodies generated to inhibit virulence traits of *E*. *coli* 0157:H7 *in vivo*.

## Materials and Methods

### Animals

Eighteen four-month-old conventionally reared Holstein Friesian male calves were obtained from a dairy farm in Buenos Aires Province, Argentina and housed at the Instituto Nacional de Tecnología Agropecuaria (INTA) research centre. Animals were selected on the basis of absence of *E*. *coli* O157:H7 by enrichment of rectoanal mucosal swabs followed by immunomagnetic separation following manufacturer`s instructions (Dynabeads anti-*E*.*coli* O157, Invitrogen Dynal AS, Oslo, Norway), and low levels of serum specific antibodies against IntiminC_280_ and EspB.

Calves were fed alfalfa pellets, with free access to hay and water and treated prophylactically upon arrival with 1% ivermectin to control intestinal nematodes. All animal experiments were performed with the approval of the Institutional Animal Care and Use of Experimentation Animals Committee (CICUAE) of the National Institution of Agricultural Reserach.

### Production of recombinant E. coli O157:H7 proteins

The coding sequence of EspB and IntiminC_280_ from the bovine *E*. *coli* O157:H7 strain 146N were cloned in pRSET-A vector (Invitrogen Corp., Carlsbad, CA) and expressed in *E*. *coli* BL21 (D3)/pLysS, as described previously [29]. Briefly, the amino terminal-His-tagged proteins were purified from the lysates by affinity chromatography on Nickel-agarose columns (ProBond nickel-chelating resin; Invitrogen Corp.), eluted under denaturing conditions and dialyzed against PBS at pH7.4.

The B subunit of Shiga toxin 2 was cloned upstream to the Brucella Lumazine Synthase (BLS) gene and the recombinant protein was expressed as described elsewhere [39]. This antigen was kindly provided by Fernando Goldbaum from Fundación Instituto Leloir.

### Immunization protocol

Calves were randomly separated into four groups and vaccinated according to the following scheme: non-vaccinated control (n = 4): PBS; Group 3Ag (n = 6): IntiminC_280_ + EspB + BLS-Stx2B; Group 2Ag (n = 6): IntiminC_280_ + EspB; Group Stx (n = 2): BLS-Stx2B. The immunization protocol consisted on two doses 15 days apart, with 100 μg of each antigen by intramuscular route. The antigens were diluted in 1 mL of PBS and emulsified in 1 mL of mineral oil-based adjuvant (Montanide ISA206, Seppic, France). The control group was vaccinated only with PBS emulsified in the adjuvant.

### Sample collection

Blood samples were collected from all animals at days 0, 9, 15 and 23 day post vaccination. Sera was separated by centrifugation at 5000 rpm for 10 min and stored at -20°C until it was used.

### Antibody response

All sera samples taken before and during the experiment were analysed by ELISA to detect specific antibodies against the antigens used in the immunization protocol, as described elsewhere [27, 39]. Briefly, 96-well Nunc-Immuno MaxiSorp assay plates (Nunc, Roskilde, Denmark) were coated overnight at 4°C with 100 μL of either IntiminC_280_ or EspB at 1 μg/mL in carbonate buffer pH 9.6 or 100 μL of Stx2B at 5 μg/mL. The latter consisted of the B subunit with a small fragment of the A subunit of Stx2 [39].

After three washes with PBS pH 7.4 containing 0.05% Tween 20 (PBST), non-specific binding sites were blocked with 3% skimmed milk in PBS for 1 hour at 37°C. After that, washes were repeated and serial two-fold dilutions of sera in PBS-T were added (100 μL/well), starting from 1/50 to assess antibodies against IntiminC_280_ and EspB. Plates were incubated for 2h at 37°C. For each plate, two wells were incubated with PBS-T alone (negative control), and a known positive sample was included. Each sample was analyzed in duplicate. After washing with PBS-T, wells were incubated for another hour with 100 μL of rabbit anti-bovine IgG1 or IgG2 conjugated with horseradish peroxidase (Bethyl Laboratories, Montgomery, USA), at dilutions of 1:10000 in PBS-T. Plates were washed three times with PBS-T. Finally, ABTS [2,2-azino-di (3-ethyl-benzthiazoline sulphonic acid)] (Amresco, Solon, USA) in citrate-phosphate buffer pH 4.2 plus 0.01% H2O2 (100μL/well) was added. Reactions were stopped after 10 min with 100 μL/well of 5% SDS and read at 405nm (OD405) in a BioTekELx808 microplate reader (BioTek Instruments, Winooski, USA). The antibody titre was expressed as the reciprocal of the end-point dilution resulting in an OD405 above the cut-off value. The cut-off value was calculated as the average plus three times the standard deviation of a negative control without serum. Plates coated with Stx2B were incubated with sera diluted 1/50 and the colour reaction was developed using OPD (o-phenylenediamine dihydrochloride). Antibodies against the B subunit of Shiga toxin 2 were expressed as OD 492.

### Functional assays

#### Inhibition of bovine red blood cell lysis

For this assay the *E*. *coli* O157:H7 438/99 strain was used. This strain was isolated from a healthy cow from a dairy farm in Buenos Aires, Argentina and is spontaneously resistantto nalidixic acid. It possesses the genes for enterohemolysin, γ-intimin, EspA, EspB, Stx2, and the pO157 plasmid.

Briefy, E. *coli* O157:H7 438/99 was grown in LB broth overnight at 37°C without shaking. OD600 was taken to measure the number of bacteria, and then diluted 1:100 into Dulbecco's modified Eagle medium (DMEM) lacking phenol red (Gibco-BRL). 2 mL of bacteria was mixed with pooled sera from each immunization group (inactivated 30 min at 56°C) to achieve a 1:20 dilution, into 12-well plates. The plates were incubated for 1 h under a 5% CO_2_ atmosphere to allow the interaction between specific antibodies and bacteria. Samples were analysed in duplicate and a positive control without sera was included.

In turn, red blood cells (RBCs) were separated by centrifugation from fresh defibrinated sheep blood, which was obtained by jugular vein punction, washed three times with 10 mM PBS (pH 7.4) and resuspended at 5% in PBS. Then, 2 ml of the 5% suspension of RBC in PBS was added to the plates and incubated for 4 h at 37°C under a 5% CO2 atmosphere. The suspension was removed from the wells and centrifuged at 12,000×*g* for 1 min. Supernatants were monitored for the presence of released haemoglobin by measuring the OD at 543 nm.

OD543 obtained from the positive control, consisting on *E*. *coli* O157:H7 strain 438/99 with no sera, was considered as 100% haemolysis and inhibition of haemolysis was expressed as 100% -% haemolysis of each treatment.

#### Inhibition of *E*. *coli* O157:H7 adherence to Hep-2 cells

12-well plates were inoculated with 2,5 X 105 cells per well of HEp-2 (ATCC-CCL-23) and grown to 60–70% confluence at 37°C in 5% CO2, in DMEM with 10% heat-inactivated foetal bovine serum, 2mM L-glutamine, penicillin (100,000 IU/liter), and streptomycin (100 mg/liter).

*E*. *coli* O157:H7 438/99 was grown in Brain Heart Infusion broth (BHI) with NaHCO_3_ 44mM overnight at 37°C without shaking. 20 μl of the overnight culture were added to 300 μl of adherence media (DMEM, mannose 1%, NaHCO_3_ 0.4%) containing pooled sera from each immunization group (inactivated 30 min at 56°C) at 1:50 dilution. A positive control without sera was also included. The mixture was incubated at 37°C for 30 min to allow the interaction between *E*. *coli* O157:H7 and the antibodies.

Hep-2 cells were washed three times with adherence media, replenished with the mixture of bacteria and sera (300 ul/well) and incubated at 37°C in 5% CO2 for three hours. After that, the cells were washed twice with PBS and replenished with DMEM plus the corresponding sera diluted 1:50. After two more hours of incubation, the infected monolayers were washed three times with PBS and the adherent bacteria were recovered with 1 mL of 0.1% Triton X100 in PBS buffer. Dilutions were plated on Sorbitol McConkey agar plates.

The number of recovered bacteria from the positive control (without sera) was considered as 100% of adherence.

#### Inhibition of Stx2 cytotoxicity on vero cells

For the neutralization assay, Stx2 (Phoenix Laboratory, Boston, MA, USA) diluted in Minimum Essential Medium (MEM; Gibco; Life Technologies Corp., Carlsbad, CA) at a concentration able to inhibit cell viability by 50% was incubated with several dilutions of pooled sera from the different immunization groups for 1 h at 37°C with shaking. These mixes were then assayed in Vero cell culture by using the neutral red assay adapted from a previously described protocol[41]. Briefly, Vero cells were plated in 96-well plates and grown to confluence in complete MEM medium. Then, cells were incubated under growth-arrested conditions either with Stx2 alone or with the sera for 72 h and the neutral red uptake was determined. Results are expressed as percentage of cell viability and 100% represents cells incubated under identical conditions but without the toxoid treatment.

### Data analysis

Data are presented as mean ± SEM of three independent experiments. Plotting and statistical analysis of data was accomplished using GraphPad Prism 5.0 (GraphPad, USA). For ELISA, RBC lysis and Hep-2 adherence, statistical analysis was performed using 1-way ANOVA and the Tukey`s Multiple Comparison post test.

Comparisons between inhibition of Stx2 cytotoxicity means of different groups was performed using two-way ANOVA. Significance was determined using Tukey`s Multiple Comparison test as a posteriori test. In all cases, statistical significance was set at P< 0.05.

## Results

### Specific IgG antibody titres increase in calves vaccinated with type three secretion system antigens and the B subunit of shiga toxin

**Fig 1:**
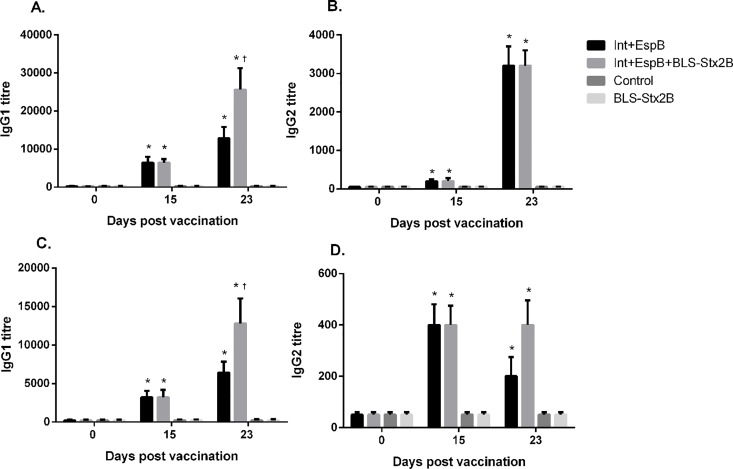
Immunoglobulin G1 (A,C) and G2 (B,D) responses to IntiminC_280_ (A, B) and EspB (C,D) in calves vaccinated with IntiminC_280_ + EspB + BLS-Stx2B (3Ag); IntiminC_280_ + EspB (2Ag); BLS-Stx2B and PBS (control) measured by ELISA at different times post vaccination. Data are shown as titers (as reciprocal of dilutions) ± standard error of the mean. * Indicates statistical significance (p < 0.05) compared to the control and † indicates statistical significance compared to the 2Ag group.

Overall, similar response patterns were observed against IntiminC_280_ and EspB although the titres against the former were higher than those observed for EspB (Fig 1). For both antigens, IgG2 titres were lower than that of IgG1and the IgG2 response to EspB drops after the booster.

The inclusion of the BLS-Stx2B in the formulation seems to have a significant stimulatory effect on the IgG1 response to IntiminC_280_ and EspB, since a significantly higher title against both antigens was observed in the group of calves vaccinated with the 3 antigens. The IgA titers to neither of these two antigens significantly increased after vaccination.

The specificity of antibodies was confirmed by Western blot (data not shown).

**Fig 2:**
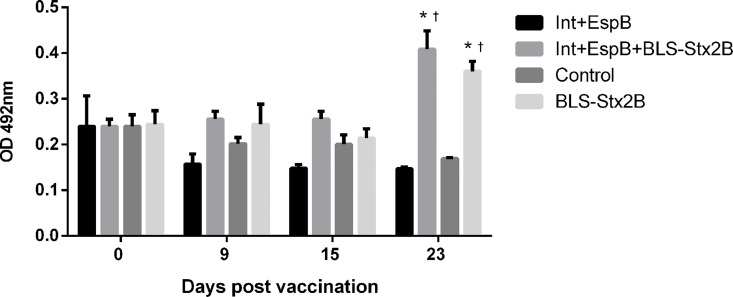
Immunoglobulin G responses to Stx2B in calves vaccinated with IntiminC_280_ + EspB (2Ag); IntiminC_280_ + EspB + BLS-Stx2B (3Ag); BLS-Stx2B and PBS (control) measured by ELISA at different times post vaccination. Data are shown as OD492 ± standard error of the mean. * Indicates statistical significance (p < 0.05) compared to the control and † indicates statistical significance compared to the preimmune condition.

The serological response against Stx2 was measured by ELISA and Fig 2 shows that the IgG response to BLS-Stx2B was low after the first dose and increased after the booster in both groups that received BLS-Stx2B (Fig 2), being statistically significant on day 23 post vaccination compared to the preimmune condition. Furthermore, the level of specific antibodies on day 23 was also significantly higher when the chimera vaccinated groups where compared to the control No difference was observed between the group vaccinated with three antigens or with BLS-Stx2B alone.

### Bovine sera from animals vaccinated with BLS-Stx2B neutralize Stx2 cytotoxicity on vero cells

**Fig 3:**
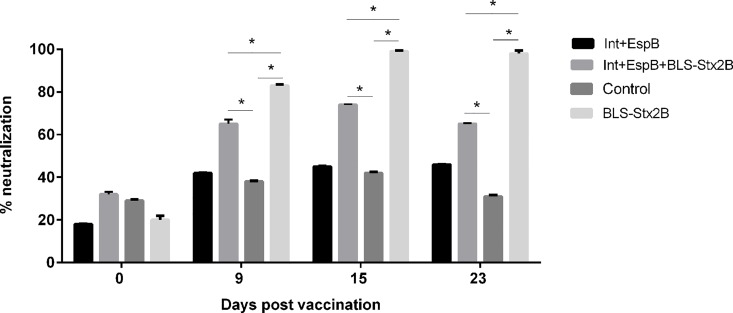
Stx2 cytotoxicity neutralization in Vero cells. Results are expressed as percentage of cell viability and 100% represents cells incubated under identical conditions but without the toxin treatment. Data are shown as mean ± standard error of the mean of 3 experiments performed in quadruplicate (*P<0.05).

Results of neutralization of cytotoxicity on Vero cells are shown in Fig 3. Sera from animals vaccinated with 3 antigens as well as sera from calves that received only BLS-Stx2B induced a significant neutralization of Stx2 cytotoxicity compared to the control group from day 9 post vaccination. As expected, the neutralization ability of sera from animals vaccinated with 2 antigens was similar to the controls. Furthermore, a statistically significant difference was observed between both groups immunized with the chimera, being the group vaccinated only with BLS-Stx2B the one that resulted in higher neutralization. A maximum neutralization of around 100%, was observed in the latter, on 15 and 23 days post vaccination.

### Vaccination elicits antibodies able to partially inhibit erythrocyte lysis induced by the T3SS

**Fig 4:**
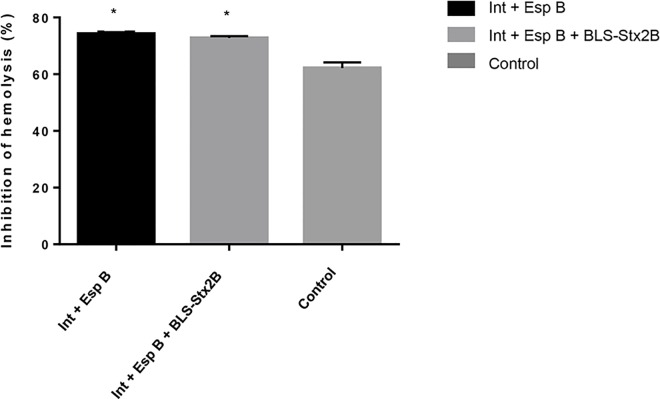
Inhibition of RBC lysis by sera from calves vaccinated with IntiminC_280_ + EspB + BLS-Stx2B (3Ag); IntiminC_280_ + EspB (2Ag); and PBS (control). Results are represented as % of inhibition of RBC lysis caused by the strain EHEC 438/99 without sera. Data are shown as mean ± standard error of the mean of 3 experiments performed. * Indicates statistical significance compared to the control (p < 0.05).

To measure the neutralizing activity of antibodies against EspB, RBC lysis was used as a correlate of T3SS activity since this protein is a component of the T3SS translocon. Both sera from animals vaccinated with 2Ag or 3Ag, inhibited RBC lysis by 74% and 73% respectively, compared to 60% of sera from non-vaccinated calves (Fig 4). This difference is statistically significant* (p < 0.05).

### Vaccination elicits antibodies able to neutralize adherence of EHEC O157:H7 to epithelial cells *in vitro*

**Fig 5:**
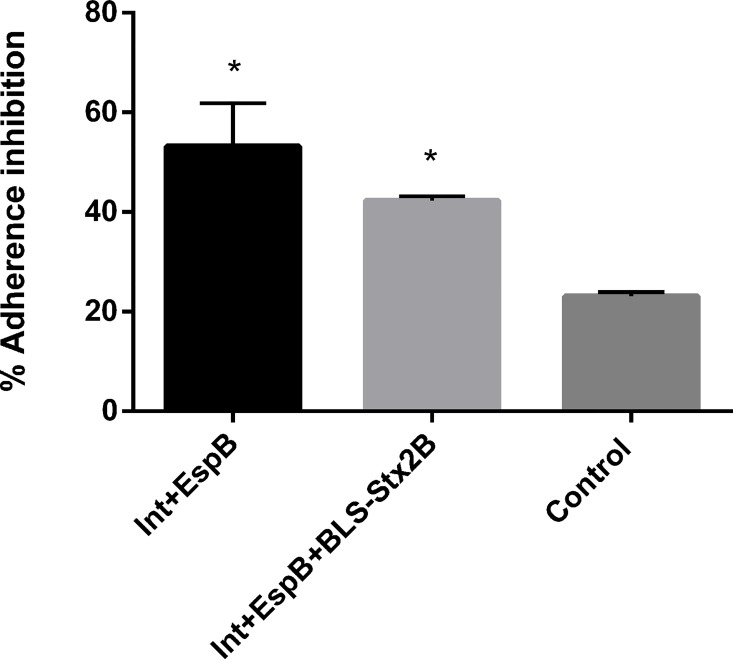
Inhibition of E. *coli* O157:H7 adherence to Hep-2 cells by sera from calves vaccinated with IntiminC_280_ + EspB + BLS-Stx2B (3Ag); IntiminC_280_ + EspB (2Ag); and PBS (control). Results are represented as % of inhibition of adherence respect to strain EHEC 438/99 without sera. Data are shown as mean ± standard error of the mean of 3 experiments performed (*P<0.05). * Indicates statistical significance compared to the control (p < 0.05).

Inhibition of *E*. *coli* O157:H7 adherence to Hep-2 cells was carried out to measure the neutralizing activity of antibodies raised through immunization. Sera from animals vaccinated with either 2 or 3 antigens significantly reduced EHEC adherence (both P<0.05) compared to sera from non-vaccinated animals, but no significant difference was observed between sera from calves vaccinated with either 2 or 3 antigens(Fig 5).

## Discussion

Vaccination is one of the pre-slaughter interventions that can be used to reduce EHEC O157:H7 occurrence in cattle [42]. In order to prevent intestinal colonization, vaccines need to target molecules involved in EHEC adherence and for this matter, Type III Secreted proteins are attractive targets for vaccine development [29, 32, 33].

In this work we have proven that vaccination of 4-month-old Fresian Holstein calves with IntiminC_280_, EspB and the fusion protein of Shiga toxin 2 B subunit and Brucella Lumazine Synthase is able to induce a significant immune response against these antigens. These results coincide with previous reports from our group, as well as with several other authors who have used T3SS proteins as antigens [23, 29, 34].

The potential of Intimin as a subunit vaccine was suggested following observations that antibodies against the carboxy-terminal domain inhibit bacterial adherence [43–46]. Furthermore, Intimin has strong antigenic determinants and it has been shown to be a target of humoral immune responses in different host species and animal models such as humans [47, 48], cattle [21, 29, 32, 34, 35], rabbits [49], pigs [50] and mice [51]. Antibodies against this protein have also been described after experimental (Bretschneider et al.[20] as well as natural infections [21, 27, 52].

On the other hand, a humoral response against EspB has been observed after vaccination, as mentioned previously, and in cattle after natural [21, 27, 52] as well as experimental infections [27, 29, 53] and in human patients suffering from HUS [53], although it is less antigenic than Intimin.

The antibodies obtained through immunization in this trial are able to inhibit several virulence traits of *E*. *coli* O157:H7 *in vitro*, as assessed in different functional assays that are a correlate of EHEC virulence. Bovine RBC lysis provoked by the T3SS was significantly neutralized by the antibodies in sera from animals vaccinated with the combination of two as well as three antigens. This neutralization is most likely caused by anti EspB antibodies as Intimin should not be involved in RBC lysis [54]. On the other hand, sera from both group of animals vaccinated with IntiminC_280_ also neutralized *E*. *coli* O157:H7 adherence to epithelial cell lines. In both cases, anti-Intimin and anti-EspB antibodies contribute to the neutralization as both virulence factors are needed for a strong attachment of EHEC to epithelia. No significant differences were observed between the neutralizing activities of sera from animals vaccinated with two and three antigens suggesting that anti Stx2B antibodies do not contribute to the inhibition of RBC lysis nor the neutralization of adherence, as expected.

Despite the low level of anti EspB and anti Intimin antibodies detected in the control group, some neutralization of adherence as well as RBC lysis was observed in the sera of these calves. This effect might be explained taking into account that many factors present in bovine serum can unspecifically inhibit the T3SS, such as lactoferrin [55] or α1-antitrypsin [56], both present in bovine serum [57].Sera from the animals vaccinated with 3 antigens contained higher titers of anti Intimin antibodies than animals vaccinated with 2 antigens; however the neutralizing activities of RBC lysis and Hep-2 cells adherence were of similar magnitude. This lack of correlation between the antibody level and neutralizing activity may be explained considering that other adhesins, not neutralized by anti Intimin antibodies, may be involved in EHEC adherence to epithelial cells [58], and that, at the dilution used in these assays, the anti Intimin antibodies present in sera from calves vaccinated with two antigens saturates all Intimin at the surface of EHEC.

As regards the immune response elicited against Stx2, we observed a specific humoral response in both groups vaccinated with BLS-Stx2B. The enzyme Lumazine Synthase from Brucella spp. (BLS) is a highly immunogenic and stable dimer of pentamers and a scaffold with enormous plasticity to display foreign antigens on its structure [59], which provides a display platform for Stx2B, since several studies have shown that its pentameric arrangement is only marginally stable [60], explaining, at least in part, its lack of immunogenicity when employed alone.

The magnitude of the IgG response is considerably lower compared to that obtained in mice with the same BLS-Stx2B chimera [39] or with other heterologous antigens carried by BLS [61–63]. Several factors might account for this difference such as the species as well as the amount of recombinant protein per weight of animal. Despite the low level of anti-Stx2B antibodies elicited, they were able to neutralize the cytotoxicity produced by Stx2 on Vero cells. Furthermore, neutralization was observed at days 9 and 15 after vaccination, even no significant increase in DO was observed. Similar results have been previously reported in mice immunized with this chimera, which were able to survive intravenous challenge with the toxin despite a low level of anti Stx2B antibodies [39], suggesting that high afinity antibodies might account for this protection. However, affinity was not assessed on this study.

Higher doses of the chimera must be tested in further studies in order to increase the level of specific antibodies against Stx2B.

Humoral immune response against Shiga toxins in cattle has also been described in natural infections. Calves bearing maternal and acquired antibodies against Stx1 and in lesser extent, Stx2 [64] have been reported, sometimes with high frequency of reacting animals [65]. These antibodies have also been detected in natural bovine colostra [66] and in cows immunized with Stx2 [22, 23]. No anti Stx2 response was observed after experimental infection of calves with *E*. *coli* O157:H7 [16, 19, 27, 67].

Furthermore, the inclusion of BLS-Stx2B in the formula induces not only antibodies against Stx2, but also potentiates the IgG1 response to Intimin and EspB. This effect may be due to BLS since this protein has a demonstrated adjuvant power [62, 68, 69].

We have previously tested a candidate vaccine consisting on the carboxy-terminal fraction of Intimin γ and EspB that resulted in reduced fecal shedding of *E*. *coli* O157:H7 after challenge[29]. In order to increase the level of protection conferred by vaccination with these two antigens, we assayed the immunogenicity of the BLS-Stx2B chimera in cattle and the ability of the antibodies elicited to neutralize Stx2 cytotoxicity. The results obtained here allow us to conclude that the chimera is a potent immunogen able to elicit Stx2 neutralizing antibodies *in vitro* and to induce a strong activation of the humoral response to Intimin and EspB.

Further studies are required to determine if the antibodies elicited against Stx2 and the further stimulation of the response to the previously assayed antigens, provides additional protection against infection of calves with E. *coli* O157:H7 than the observed for Intimin and EspB alone. Also the relevance of T-cell mediated immunity was recently demonstrated suggesting the existence of unexplored epitopes in Intimin that may provide new clues for a stronger protective response [67].

These results presented here bring insight into vaccine development to prevent bovine carriage of *E*. *coli* O157:H7 thus, providing a tool to control a national health issue as HUS.
